# One-year health-related quality of life outcomes in weight loss trial participants: comparison of three measures

**DOI:** 10.1186/1477-7525-7-53

**Published:** 2009-06-09

**Authors:** Ronette L Kolotkin, Josephine M Norquist, Ross D Crosby, Shailaja Suryawanshi, Pedro J Teixeira, Steven B Heymsfield, Ngozi Erondu, Allison M Nguyen

**Affiliations:** 1 Obesity and Quality of Life Consulting, 762 Ninth Street #563, Durham, North Carolina 27705, USA; 2 Department of Community and Family Medicine, Duke University Medical Center, 318 Hanes House, Box 2914, Durham, North Carolina 27710, USA; 3 Merck Research Laboratories, UG1D-60, PO Box 1000, North Wales, Pennsylvannia 19454, USA; 4 Neuropsychiatric Research Institute, 120 Eighth Street South, PO Box 1415, Fargo, North Dakota 58107, USA; 5 University of North Dakota School of Medicine and Health Sciences, 1919 Elm Street North, Room 118, Fargo, North Dakota 58102, USA; 6 Merck Research Laboratories, 126 E Lincoln Avenue, Rahway, New Jersey 07065, USA; 7 Faculdade de Motricidade Humana, Estrada da Costa, 1495-688 Cruz Quebrada, Portugal

## Abstract

**Background:**

The literature on changes in health-related quality of life (HRQOL) in weight loss studies is inconsistent, and few studies use more than one type of measure. The purpose of the current study was to compare one-year changes in HRQOL as a function of weight change using three different measures: a weight-related measure (Impact of Weight on Quality of Life-Lite [IWQOL-Lite)]) and two generic measures (SF-36; EQ-5D).

**Methods:**

Data were obtained from 926 participants (mean Body Mass Index (BMI) (kg/m^2^) = 35.4; 84% female; mean age = 49.5 years) in a placebo-controlled randomized trial for weight loss. At baseline and one-year, participants completed all three HRQOL measures. HRQOL was compared across weight change categories (≥ 5% and 0–4.9% gain, 0–4.9%, 5.0–9.9% and ≥ 10% loss), using effect sizes.

**Results:**

The weight-related measure of HRQOL exhibited greater improvements with one-year weight loss than either of the generic instruments, with effect sizes ranging from 0.24 to 0.62 for 5–9.9% weight reductions and 0.44 to 0.95 for ≥ 10% reductions. IWQOL-Lite Self-Esteem also showed a small improvement with weight gain. Changes in the two generic measures of HRQOL were inconsistent with each other, and in the case of the SF-36, variable across domains. For participants gaining ≥ 5% of weight, the greatest reductions in HRQOL occurred with respect to SF-36 Mental Health, MCS, and Vitality, with effect sizes of -0.82, -0.70, and -0.63 respectively.

**Conclusion:**

This study found differences between weight-related and generic measures of health-related quality of life in a one-year weight loss trial, reflecting the potential value of using more than one measure in a trial. Although weight loss was generally associated with improved IWQOL-Lite, physical SF-36 subscale and EQ-5D scores, a small amount of weight gain was associated with a slight improvement on weight-specific HRQOL and almost no change on the EQ-5D, suggesting the need for further research to more fully study these relationships. We believe our findings have relevance for weight loss patients and obesity clinicians/researchers in informing them of likely HRQOL outcomes associated with varying amounts of weight loss or gain.

## Background

There is growing interest in assessing patient-reported outcomes (PRO) in clinical trials along with more traditional clinical primary endpoints. One type of PRO, health-related quality of life (HRQOL), may be assessed using either generic measures that are applicable to any population or measures specific to the disease under study. In the weight loss literature, HRQOL outcomes have been reported using both types of measures.

Generic and disease-specific measures of HRQOL each have their advantages and disadvantages. Generic measures are applicable to any population and scores may be compared to general population norms as well as across diseases. Disease-specific measures contain items of particular relevance to patients with the disease, and as such, have inherent face validity and salience. Additionally, disease-specific measures have the potential to be more sensitive to smaller differences between groups and smaller changes over time than generic measures, because of their specificity [1].

A meta-analysis [2] of 54 cross-sectional studies of obese persons seeking or not seeking various weight loss treatments focused only on studies that used the generic Medical Outcomes Study Short Form-36 (SF-36) [3] or the weight-related Impact of Weight on Quality of Life-Lite (IWQOL-Lite) [4]. These authors found larger differences among populations (i.e. general population, non-treatment-seeking obese, conservative-treatment-seeking obese, and bariatric surgery patients) for the IWQOL-Lite than the SF-36. After adjusting for weight, the population differences disappeared for the IWQOL-Lite, but remained for the SF-36. In our own work with over 10,000 subjects who have taken the IWQOL-Lite, differences across populations remain after controlling for BMI [5]. Differences between our findings and those from the van Nunen et al meta-analysis could be partially due to differences in statistical methods. That is, the unit of analysis in a meta-analysis is the study, whereas the unit of analysis in a single study is the individual.

A review of HRQOL outcomes in 34 randomized controlled trials for weight loss interventions indicated inconsistencies across studies, with varying types of measures used, diverse assessment points, and differing outcomes [6]. Even when the same measure was used – for example, the SF-36 – positive treatment effects were shown for some domains, but not others, and these domains varied across studies. When obesity-specific measures were used, a greater percentage of the trials showed improved HRQOL [6]. Since nearly all the weight loss trials used only one type of HRQOL instrument, the opportunity to compare changes in generic vs. obesity-specific measures within a single trial was limited. In a 4-month trial Kaukua and colleagues [7] administered both the SF-36 and an obesity-specific measure [Obesity-Related Psychosocial Problems scale (OP Scale) [8]] to a group of men randomized to a very-low-energy diet plus behavior modification or a wait list control group. Improvements on two of the SF-36 domains (physical and social functioning) and the OP Scale were maintained until the end of follow-up for the treated subjects. In a case-controlled study by Karlsson et al [9], both generic (Sickness Impact Profile) and obesity-specific (OP Scale) HRQOL improved after gastric restriction surgery. However, changes in the OP Scale were also significant for women, but not men, in the control group.

Thus, the current literature on changes in HRQOL in weight loss studies is inconsistent, and few studies use more than one type of measure within a single study. The purpose of the current study was to compare HRQOL outcomes over a one-year time period as a function of weight change in a weight loss clinical trial using three different measures of HRQOL: IWQOL-Lite, SF-36, and EQ-5D [10]. We are aware of no other studies that used these particular measures in a single weight loss trial.

## Methods

### Participants

Data were obtained from adults who participated in a one-year randomized, placebo-controlled trial of an experimental medication for weight loss (trial number PN011). Quorum Review, Inc. (Seattle, Washington), an independent institutional review board, approved the study, and informed consent was obtained from all participants. All research was conducted in compliance with the Helsinki Declaration. For inclusion in the trial patients had to be at least 18 years old and meet the following criteria: body mass index (BMI) between 30–43 kg/m^2^, or BMI ≥ 27 kg/m2 with obesity-related comorbidities, stable weight for three months prior to screening, and the ability to read and understand questionnaires. All participants providing baseline and year-one HRQOL data were included in the present analysis.

### Procedures

Full details of the study have been described previously (e.g. recruitment, retention, institutional review) [11]. Prior to randomization there was a two-week diet and exercise run-in during which patients were administered placebo in a single-blinded manner. Eligible participants were then randomized in a 2:1 ratio to either the experimental drug group or the placebo group. The patients were instructed to follow a diet with a 500 kcal/day deficit in caloric intake, and they received dietary and exercise counseling. At baseline and at one-year follow-up, patients completed HRQOL instruments as described below.

### Measures

#### Impact of Weight on Quality of Life-Lite (IWQOL-Lite)

The IWQOL-Lite is a validated 31-item, self-report measure of weight-related quality of life that provides a total score plus scores on five domains (Physical Function, Self-Esteem, Sexual Life, Public Distress, and Work) [4]. The IWQOL-Lite has been shown to have good internal consistency (ranging from .90 to .96) [4], good test-retest reliability (.83 to .94) [12], responsiveness to weight loss and weight gain [13,14], sensitivity to treatment-seeking status [15,16] and degree of obesity [17], and a scale structure supported by confirmatory factor analysis [4]. Scores are transformed to a 0 to 100 scale, where 100 represents the best HRQOL.

#### Medical Outcomes Study Short Form Health Survey (SF-36, Version 2.0)

The SF-36 is a widely used survey instrument for assessing a patient's generic HRQOL [3]. It has been validated in numerous diseases and used across the world as an indicator of a patient's perception of his or her current health status. The SF-36 provides scores on eight domains: Physical Functioning, Role Physical, Bodily Pain, General Health, Vitality, Social Functioning, Role Emotional, and Mental Health. In addition, two component scores can be calculated: the Physical Component Summary (PCS) and Mental Component Summary (MCS). Scores are transformed to a 0 to 100 scale, where 100 represents the best HRQOL.

#### EQ-5D

The EQ-5D is a utility instrument that may be used as a generic measure of health outcome. Applicable to a wide range of health conditions and treatments, it provides a simple descriptive profile and a single index value for health status that can be used in the clinical and economic evaluation of health care, as well as population health surveys. The EQ-5D has been specially designed to complement other quality of life measures such as the SF-36 or disease-specific measures. [18]. It is composed of five questions assessing attributes including mobility, self-care, usual activities, pain/discomfort, and anxiety/depression. .

### Statistical Analyses

Data from the experimental drug group and placebo group were pooled for the analyses. This approach was warranted because no clinically meaningful differences in efficacy or safety were observed between groups in previously published analyses [11]. Descriptive statistics (mean, SD) for the HRQOL measures were calculated at baseline and week 52. Changes in HRQOL over the 52-week period were calculated as the difference between baseline and week-52 scores. Patients were categorized according to the percent of weight change observed during this interval (≥ 5% gain, 0–4.9% gain, 0–4.9% loss, 5–9.9% loss, and ≥ 10% loss). The purpose of using weight change categories rather than weight as a continuous variable was to facilitate clinical relevance and interpretation of the findings. Effect size statistics for each group were calculated by dividing the 52-week mean change score by the standard deviation of the corresponding baseline score. Estimates of 0.2, 0.5 and 0.8 were considered small, moderate and large, respectively [19]. For each measure and weight change category, mean change in domain scores from baseline to one-year were calculated and compared to a reference group using analysis of covariance controlling for baseline scores. For this analysis, the 0–4.9% weight loss category was chosen as the reference category because weight loss above that threshold (i.e., ≥ 5%) is considered a meaningful change from a clinical perspective [20].

## Results

The sample used in these analyses consisted of 926 patients (931 had completed the original 1-year trial [11]; five patients had incomplete HRQOL data and were dropped from the current analyses). Of these 926 patients, 779 (84%) were women and 727 (79%) were white. The mean (SD) age in years was 49.5 (11.1) with a range of 20 to 78 years (Table 1). The average 52-week weight loss was 2.7% (SD = 6.6, range -28.8% to 21.2%). The frequencies (%) in the five weight change categories were:

**Table 1:** Participant Baseline Characteristics (n = 926)

	**n (%)**
**Gender**	
Female	779 (84%)
Male	147 (16%)

**Race**	
White	727 (78.5%)
Black	108 (11.7%)
Hispanic	72 (7.8%)
Asian	14 (1.5%)
Other	5 (0.5%)

**Age **(years) [Mean (SD)]	49.5 (11.1)

**BMI **(kg) [Mean (SD); range]	35.4 (3.8); 27.3–44.1

• ≥ 5% gain: 79 (8.5%)

• 0 to 4.9 gain: 243 (26.2%)

• 0 to 4.9% loss: 323 (34.9%)

• 5 to 9.9% loss: 168 (18.1%)

• ≥ 10% loss: 112 (12.1%)

**Figure 1:**
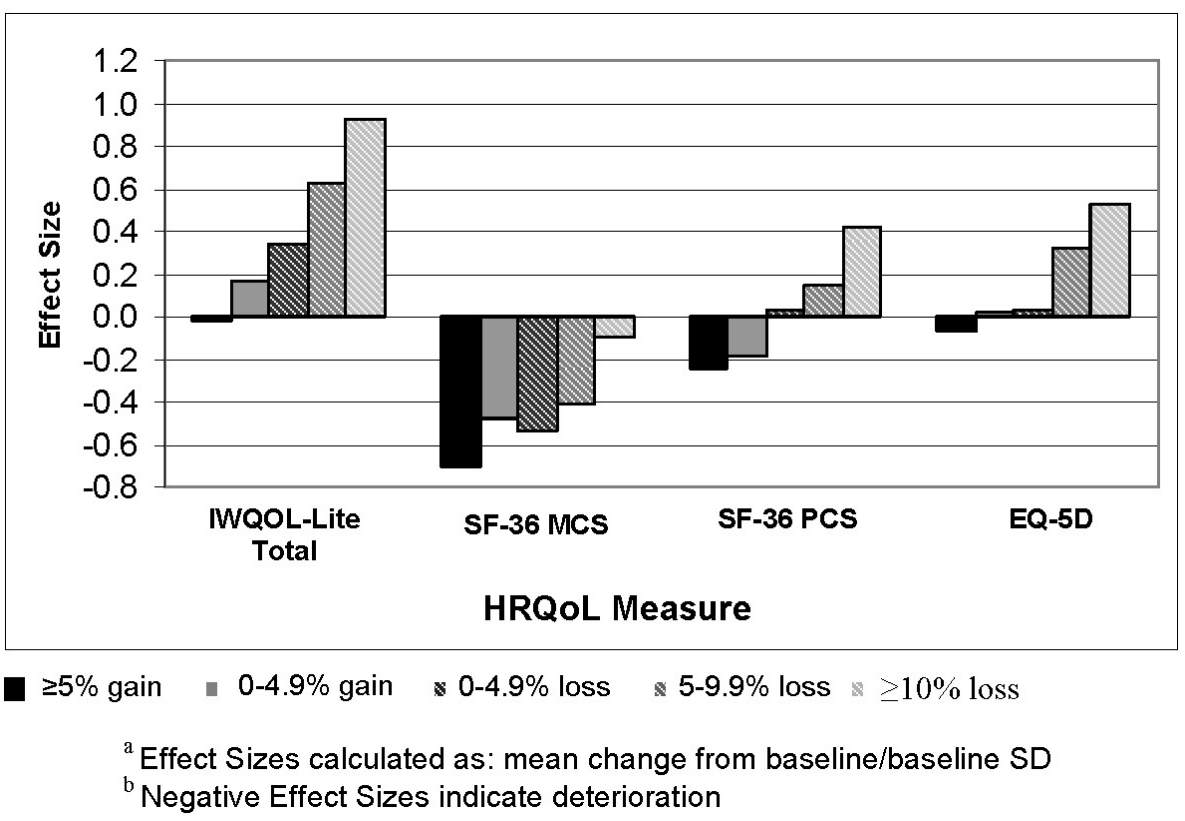
**Effect sizes by category of weight change**.

Baseline and mean one-year changes in IWQOL-Lite scores by weight change category, and corresponding effect sizes, are summarized in Table 2. Similar statistics for the EQ-5D domains and the SF-36 are summarized in Tables 3 and 4, respectively. For both the IWQOL-Lite and the EQ-5D, improvements in HRQOL scores were observed in an increasing trend across the weight change categories, although effect sizes for the IWQOL-Lite were much larger (Figure 1). On the IWQOL-Lite a 5.0–9.9% weight loss was associated with moderate effect sizes on Physical Function, Self-Esteem, and total score (0.57, 0.58, and 0.62, respectively), and a ≥ 10% weight loss was associated with large effect sizes on these domains (0.95, 0.95, and 0.93, respectively). Of note, weight gain was associated with small improvements in IWQOL-Lite Self-Esteem (effect size of 0.21 for the greater than or equal to 5% weight gain category and 0.34 for the 0 to 4.9% gain category). However, no such improvements were observed with weight gain on the EQ-5 D.

**Table 2:** IWQOL-Lite Scores at Baseline and 1-Year

**IWQOL Scores by Weight loss/gain category**	**Baseline Mean (SD)**	**Change^a ^Mean (SD)**	**Effect Size^b^**
**Total Score**			
>= 5% gain (n = 79)	72.1 (16.7)	-0.4 (12.4)	-0.02
0.1–4.9% gain (n = 243)	73.9 (15.6)	2.7 (11.0)	0.17
0–4.9% loss (n = 323)	74.2 (16.2)	5.5 (10.4)	0.34
5–9.9% loss (n = 164)	74.1 (16.2)	10.0 (11.2)	0.62
≥ 10% loss (n = 111)	71.4 (17.6)	16.4 (13.7)	0.93

**Physical Function**			
>= 5% gain (n = 79)	73.5 (19.6)	-4.1 (14.0)	-0.21
0.1–4.9% gain (n = 244)	73.8 (18.7)	1.4 (13.8)	0.07
0–4.9% loss (n = 323)	74.2 (17.4)	5.6 (12.5)	0.32
5–9.9% loss (n = 167)	73.2 (19.7)	11.3 (14.5)	0.57
≥ 10% loss (n = 112)	70.5 (19.2)	18.3 (14.5)	0.95

**Self Esteem**			
>= 5% gain (n = 79)	52.0 (24.7)	5.1 (20.6)	0.21
0.1–4.9% gain (n = 243)	56.9 (24.6)	8.4 (18.9)	0.34
0–4.9% loss (n = 323)	57.6 (26.3)	9.8 (16.5)	0.37
5–9.9% loss (n = 166)	58.4 (26.2)	15.3 (18.2)	0.58
≥ 10% loss (n = 112)	56.0 (23.4)	22.3 (18.9)	0.95

**Sexual Life**			
>= 5% gain (n = 76)	76.1 (27.9)	-1.7 (20.5)	-0.06
0.1–4.9% gain (n = 238)	75.7 (25.7)	0.6 (21.3)	0.02
0–4.9% loss (n = 308)	75.3 (26.1)	5.0 (19.6)	0.19
5–9.9% loss (n = 155)	76.3 (27.7)	8.1 (20.8)	0.29
≥ 10% loss (n = 107)	74.9 (26.6)	14.2 (22.4)	0.53

**Public Distress**			
>= 5% gain (n = 79)	81.3 (19.6)	0.1 (15.9)	0.005
0.1–4.9% gain (n = 243)	85.3 (17.0)	1.3 (12.2)	0.08
0–4.9% loss (n = 323)	85.0 (18.9)	3.6 (12.9)	0.19
5–9.9% loss (n = 166)	85.8 (15.8)	6.0 (11.7)	0.38
≥ 10% loss (n = 111)	81.8 (19.1)	11.7 (17.0)	0.61

**Work**			
>= 5% gain (n = 79)	87.7 (16.1)	-0.4 (13.4)	-0.02
0.1–4.9% gain (n = 243)	88.4 (15.9)	0.2 (12.6)	0.01
0–4.9% loss (n = 321)	88.0 (15.8)	1.6 (12.4)	0.10
5–9.9% loss (n = 160)	89.3 (14.2)	3.4 (13.5)	0.24
≥ 10% loss (n = 111)	85.8 (19.0)	8.3 (18.0)	0.44

^a ^Negative changes indicate deterioration; positive changes indicate improvement

^b ^Based on standard deviation at baseline

**Table 3:** EQ-5D Scores at Baseline and 1-Year

EQ-5D Scores by Weight loss/gain category	Baseline Mean (SD)	Change^a ^Mean (SD)	Effect Size^b^
≥ 5% gain (n = 79)	79 (16.8)	-1.2 (19.5)	-0.07
0.1–4.9% gain (n = 244)	77.8 (17.5)	0.4 (16.2)	0.02
0–4.9% loss (n = 323)	79.5 (14.6)	0.4 (15.1)	0.03
5–9.9% loss (n = 164)	79.6 (14.5)	4.7 (12.6)	0.32
≥ 10% loss (n = 111)	76.8 (16.5)	8.8 (15.7)	0.53

^a ^Negative changes indicate deterioration; positive changes indicate improvement

^b ^Based on standard deviation at baseline

**Table 4:** SF-36 Scores at Baseline and 1 year

**SF-36 Scores by Weight loss/gain category**	**Baseline Mean (SD)**	**Change^a ^Mean (SD)**	**Effect Size^b^**
**Mental Component Score (MCS)**			
≥ 5% gain (n = 78)	53.6 (8.0)	-5.6 (9.5)	-0.70
0.1–4.9% gain (n = 241)	53.9 (6.9)	-3.3 (9.3)	-0.48
0–4.9% loss (n = 321)	53.7 (7.0)	-3.8 (9.0)	-0.54
5–9.9% loss (n = 162)	54.4 (6.6)	-2.7 (9.2)	-0.41
≥ 10% loss (n = 112)	53.3 (7.1)	-0.7 (7.8)	-0.10

**Physical Component Score (PCS)**			
≥ 5% gain (n = 78)	51.8 (7.1)	-1.7 (6.6)	-0.24
0.1–4.9% gain (n = 241)	51.6 (6.9)	-1.3 (7.4)	-0.19
0–4.9% loss (n = 321)	52.0 (5.9)	0.2 (6.6)	0.03
5–9.9% loss (n = 162)	51.0 (7.2)	1.1 (6.4)	0.15
≥ 10% loss (n = 112)	51.0 (6.7)	2.8 (6.8)	0.42

**Physical Functioning**			
≥ 5% gain (n = 79)	84.4 (18.4)	-4.8 (17.0)	-0.26
0.1–4.9% gain (n = 243)	84.1 (18.7	-2.2 (19.3)	-0.12
0–4.9% loss (n = 323)	85.0 (17.0)	1.4 (17.0)	0.08
5–9.9% loss (n = 165)	82.0 (19.8)	2.8 (17.0)	0.14
≥ 10% loss (n = 112)	82.5 (18.3)	8.1 (19.5)	0.44

**Role Physical**			
≥ 5% gain (n = 79)	90.0 (16.2)	-7.8 (17.9)	-0.48
0.1–4.9% gain (n = 243)	89.4 (18.4)	-4.4 (22.9)	-0.24
0–4.9% loss (n = 323)	90.8 (16.1)	-2.3 (18.8)	-0.14
5–9.9% loss (n = 165)	89.2 (17.2)	-1.0 (18.9)	-0.06
≥ 10% loss (n = 112)	89.2 (17.9)	3.7 (18.2)	0.21

**Bodily Pain**			
≥ 5% gain (n = 79)	79.4 (22.2)	-7.7 (24.4)	-0.35
0.1–4.9% gain (n = 244)	80.0 (20.4)	-7.1 (22.8)	-0.35
0–4.9% loss (n = 323)	78.5 (18.8)	-4.2 (22.5)	-0.22
5–9.9% loss (n = 164)	77.4 (20.8)	-2.2 (22.2)	-0.11
≥ 10% loss (n = 112)	77.4 (19.6)	1.0 (22.0)	0.05

**General Health**			
≥ 5% gain (n = 78)	78.0 (15.3)	-4.9 (10.7)	-0.32
0.1–4.9% gain (n = 243)	78.7 (15.2)	-4.1 (14.2)	-0.27
0–4.9% loss (n = 323)	79.8 (13.7)	-3.0 (14.2)	-0.22
5–9.9% loss (n = 166)	79.4 (14.2)	0.2 (12.4)	0.01
≥ 10% loss (n = 112)	76.9 (13.3)	4.8 (13.2)	0.36

**Vitality**			
≥ 5% gain (n = 78)	67.9 (18.6)	-11.8 (18.1)	-0.63
0.1–4.9% gain (n = 243)	66.8 18.2)	-6.9 (19.4)	-0.38
0–4.9% loss (n = 322)	66.0 (17.8)	-4.8 (17.5)	-0.27
5–9.9% loss (n = 166)	67.3 (16.1)	-0.8 (17.7)	-0.05
≥ 10% loss (n = 112)	62.9 (18.1)	5.2 (19.0)	0.29

**Social Functioning**			
≥ 5% gain (n = 79)	90.7 (17.3)	-8.7 (19.3)	-0.50
0.1–4.9% gain (n = 244)	91.2 (15.9)	-5.9 (22.8)	-0.37
0–4.9% loss (n = 323)	92.1 (15.4)	-5.6 (19.4)	-0.36
5–9.9% loss (n = 166)	93.0 (13.6)	-3.5 (19.1)	-0.26
≥ 10% loss (n = 112)	92.0 (16.7)	0.1 (20.0)	0.006

**Role Emotional**			
≥ 5% gain (n = 79)	91.5 (15.5)	-7.3 (20.4)	-0.47
0.1–4.9% gain (n = 243)	93.2 (14.9)	-6.2 (22.7)	-0.42
0–4.9% loss (n = 322)	93.3 (13.4)	-5.6 (18.1)	-0.41
5–9.9% loss (n = 163)	94.7 (11.9)	-5.7 (19.1)	-0.48
≥ 10% loss (n = 112)	94.4 (12.0)	-1.9 (15.6)	-0.16

**Mental Health**			
≥ 5% gain (n = 78)	82.6 (13.3)	-10.9 (16.6)	-0.82
0.1–4.9% gain (n = 243)	82.8 (12.9)	-5.0 (16.8)	-0.39
0–4.9% loss (n = 322)	82.2 (13.1)	-6.2 (15.8)	-0.47
5–9.9% loss (n = 166)	81.4 (13.7)	-3.1 (18.1)	-0.23
≥ 10% loss (n = 112)	79.9 (13.9)	0.4 (15.4)	0.03

^a ^Positive changes indicate improvement; negative changes indicate deterioration

^b ^Based on standard deviation at baseline

A different pattern of results emerged for the SF-36. Patients in all weight change categories showed deterioration in MCS scores, ranging from small to moderately large effects. PCS scores showed very small improvements associated with a 5–9.9% weight loss and moderate improvements for patients who experienced a ≥ 10% loss. Weight gain was associated with very small to small reductions on PCS scores, but moderate to moderately large reductions on MCS. With respect to SF-36 subscales, the greatest improvement associated with weight loss occurred on Physical Functioning, with a moderate improvement for patients losing at least 10% of their weight. Four domains of the SF-36 (Bodily Pain, Social Functioning, Role Emotional, and Mental Health) showed deterioration or no change in all weight change categories. The SF-36 subscales showing the greatest deterioration for a weight gain ≥ 5% were Mental Health and Vitality (with effect sizes of -0.82, and -0.63 respectively).

**Figure 2:**
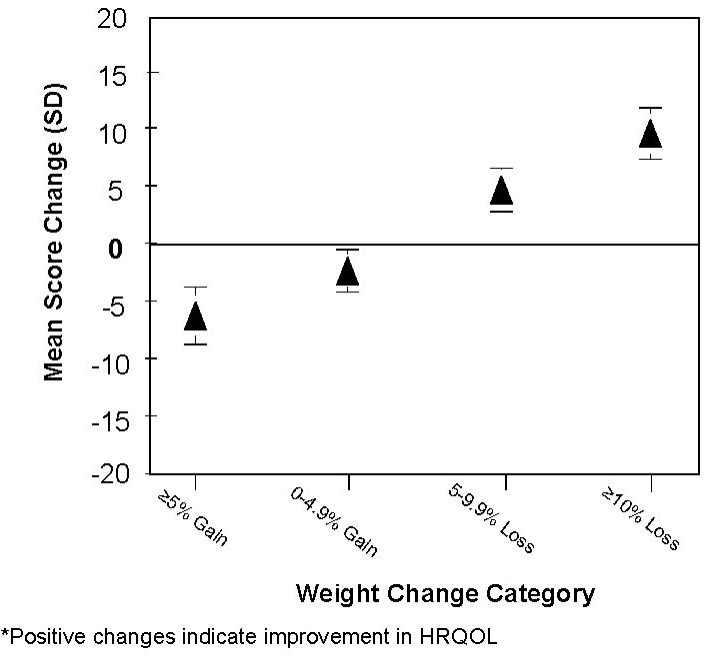
**Mean 1-Year change in IWQOL-Lite total score across weight change categories relative to the reference category (0–4.9% loss)**.

Mean subscale score changes from baseline to one-year for each weight change category were calculated for the three measures and compared to the reference category of 0–4.9% weight loss. Figure 2 shows mean score differences between each weight loss category and the reference category (0–4.9% loss) for the IWQOL-Lite total score. All group comparisons were statistically significant (p < 0.05). In other words, both weight gain categories (> = 5% and 0–4.9% gain) had negative change scores compared to the reference category, indicating deterioration in HRQOL, while both weight loss categories (5–9.9% and ≥ 10% loss) had positive change scores, indicating improvement in HRQOL over the one-year period. Similar trends were observed across all 3 HRQOL measures and domains (data not shown). However, for the SF-36, statistically significant differences (p < 0.05) were only observed between the reference group and the ≥ 10% weight loss category, except for General Health and Vitality.

## Discussion

Consistent with results of a review of randomized controlled trials for weight loss interventions [6], we observed greater improvements in the weight-related measure of HRQOL than in two generic measures in a one-year weight loss trial. Few weight loss studies have used both obesity-specific and generic measures to assess HRQOL outcomes within the same study. Kaukua and colleagues reported stronger correlations between weight change and HRQOL change for the OP Scale, an obesity-specific measure, (r = 0.635) than for SF-36 physical functioning (r = -0.502) [7].

Results of the two generic measures included in this trial were inconsistent with each other, and the subscales of the SF-36 were variable across domains. For the EQ-5D, weight loss corresponded more closely to HRQOL changes than did weight gain. Weight change (both loss and gain) seemed to correspond closely for PCS scores of the SF-36. Improvements in PCS were greater for the 10+% weight loss category than the 5–9.9% category; weight gain was associated with small reductions in PCS that were further reduced by additional weight gain. For the SF-36 subscales, weight loss was associated with improvement on some domains, but deterioration or no change on others. Variability with respect to changes in HRQOL, both within and across weight loss studies, has been reported previously [6]. Physical Functioning and PCS were most responsive to weight loss in the current study, a finding also reported in some of the weight loss trials reviewed (e.g. [7,21]). Social Functioning, Role Emotional, Mental Health, and MCS showed poor correspondence with weight change.

Few studies have explored the effects of weight gain on HRQOL. Engel and colleagues [14] found that changes in weight-related HRQOL for participants in a weight loss trial were similar in degree, but opposite in direction for weight loss and weight gain. That is, weight loss was associated with improved HRQOL and weight gain was associated with reduced HRQOL, and these changes occurred in a linear fashion. Among the individuals who gained 5% or more of their weight in our study, scores on Mental Health, MCS, and Vitality showed the greatest deterioration. Unlike the Engel et al. study, we found improved weight-related HRQOL for the group that gained 0–4.9% of their weight and only a slight decrement for the group that gained 5+% of their weight. Because no generic measures of HRQOL were used in the Engel et al. study, we cannot compare that part of our results to theirs. A prospective cohort study of 40,098 women participating in the Nurses' Health Study [22] found that women who had gained 5 pounds or more over the course of four years reported significant impairment in SF-36 Physical Functioning, Vitality, and Bodily Pain. In the present study these three domains of the SF-36, as well as several others, exhibited impairments associated with weight gain. As more of the world's population is gaining weight, a potential fruitful focus of future investigations is the effect of weight gain on HRQOL (the current literature focuses on effects of weight loss and cross-sectional differences among BMI groups).

Results of this study support the potential value of assessing HRQOL changes in weight loss trials with more than one instrument. In the best of all worlds we recommend the use of an assessment battery, the approach taken in the Swedish Obese Subjects studies [8], since each instrument contributes somewhat different information. However, we recognize the practical limitations of this approach in most clinical trials with respect to cost and respondent burden.

We believe our findings have direct relevance for weight loss patients and clinicians/researchers who work with this population. Especially if replicated in other studies of different weight loss interventions, we can use these results to inform patients and clinicians/researchers of what HRQOL changes they can expect to experience with varying amounts of weight loss or weight gain. For example, based on the current findings as well as previous findings [13] we can say with some certainty that weight loss of at least 5% is most likely to have a positive effect on weight-related physical function and self-esteem, as well as cardiovascular risk factors [20]. Knowledge of this information may serve to keep patients motivated, which as clinicians and patients are well aware, is frequently a challenge. On the other hand, knowledge of the likely adverse effects on HRQOL with increased weight may serve to reinforce the importance of weight maintenance. We know from previous research [14] that weight regain is associated with reduced weight-related HRQOL.

One of the strengths of this study is that we were able to compare HRQOL changes on three different measures of HRQOL. Although the SF-36 has been widely used in weight loss studies [6,23] and the EQ-5D has been studied with respect to BMI [24,25], this study is unique in its comparison of three measures of HRQOL outcomes in weight loss patients. Other strengths of this study include the large sample size (n = 926) and the longitudinal design with one-year follow-up assessment on all HRQOL measures. However, this study is not without limitations. The current sample was predominantly female (84%) and Caucasian (79%), limiting generalizability to other patient populations. Additionally, only a subset of the sample experienced what might be considered clinically meaningful weight loss. Only 30% of the sample lost at least 5% of their baseline weight and only 12% lost at least 10% of their baseline weight. This limits our ability to make inferences about HRQOL changes as a result of more substantial weight loss. On the other hand, having large variability in weight change, including subjects who gained weight, should increase the study's external validity. Another limitation of this study is that only 56% of the trial participants completed the one-year protocol; it is unknown in what ways attrition may limit the generalizability of our findings. In addition, it is unknown whether the results we observed would generalize to other weight loss methods and other placebo-controlled trials for different pharmacological agents. Finally, it would be an over simplification to suggest that changes in HRQOL depend solely on amount of weight change. Health care providers and clinical researchers who treat obese individuals recognize that changes in HRQOL could be influenced by a variety of variables not explored in the current study, such as initial weight loss expectations, satisfaction with weight loss results and the treatment program, self-esteem and other psychological variables, as well as comorbid health. We lack the data to address the potential role of these other variables.

## Conclusion

Because HRQOL outcomes vary with type of measure, there is potential value in using more than one instrument in studies of weight loss interventions. In a one-year weight loss trial greater improvements were found in the weight-related measure of health-related quality of life than two generic measures. There was closer correspondence between weight loss and improvements in HRQOL for the weight-related measure than the other measures, but for weight gain this was not the case. Results of the two generic measures were inconsistent with each other and, in the case of the SF-36, variable across domains. We believe the current findings may be relevant for weight loss patients and obesity clinicians and researchers in that they can be used to inform expectations regarding HRQOL and various levels of weight loss or gain.

## Abbreviations

HRQOL: Health-related quality of life; PRO: Patient reported outcomes; IWQOL-Lite: Impact of Weight on Quality of Life-Lite questionnaire; SF-36: Medical Outcomes Study Short Form-36; BMI: Body Mass Index; OP Scale: Obesity-Related Psychosocial Problems scale; PCS: Physical Component Summary; MCS: Mental Component Summary; SD: Standard deviation.

## Competing interests

RLK and RDC received consulting fees from Merck Research Laboratories for their participation in this study. RLK receives royalties from Duke University for the use of the IWQOL-Lite. JMN, SS, SBH, NE, and AMN are employed by Merck Research Laboratories, the sponsor of this study.

## Authors' contributions

RLK had the primary role in drafting the manuscript. All other authors revised the manuscript for intellectual content and gave final approval for the current version. RLK, RDC, and AMN conceived of the analysis design. RDC, JMN and AMN did the statistical analyses and had the primary role in drafting the results, tables, and figures. NE participated in the design and clinical monitoring of the weight loss study.
